# Analysis of the Material and Coating of the Nameplate of Vila D. Bosco in Macau

**DOI:** 10.3390/ma18102190

**Published:** 2025-05-09

**Authors:** Liang Zheng, Jianyi Zheng, Xiyue He, Yile Chen

**Affiliations:** Heritage Conservation Laboratory, Faculty of Humanities and Arts, Macau University of Science and Technology, Avenida Wai Long, Taipa, Macau 999078, China; zhengliang@must.edu.mo (L.Z.); jyzheng@must.edu.mo (J.Z.); hexiyue@must.edu.mo (X.H.)

**Keywords:** metal corrosion, nameplate coating, building materials, modern and contemporary architectural heritage, Portuguese architecture, Macau

## Abstract

This study focuses on the nameplate of Vila D. Bosco, a modern building in Macau from the time of Portuguese rule, and looks at the types of metal materials and surface coatings used, as well as how they corrode due to the tropical marine climate affecting the building’s metal parts. The study uses different techniques, such as X-ray fluorescence spectroscopy (XRF), scanning electron microscopy/energy dispersive spectroscopy (SEM-EDS), X-ray diffraction (XRD), attenuated total internal reflectance Fourier transform infrared spectroscopy (ATR-FTIR), and cross-sectional microscopic analysis, to carefully look at the metal, corrosion products, and coating of the nameplate. The results show that (1) the nameplate matrix is a resulfurized steel with a high sulfur content (Fe up to 97.3% and S up to 1.98%), and the sulfur element is evenly distributed inside, which is one of the internal factors that induce corrosion. (2) Rust is composed of polycrystalline iron oxides such as goethite (α-FeOOH), hematite (α-Fe_2_O_3_), and magnetite (Fe_3_O_4_) and has typical characteristics of atmospheric oxidation. (3) The white and yellow-green coatings on the nameplate are oil-modified alkyd resin paints, and the color pigments are TiO_2_, PbCrO_4_, etc. The surface layer of the letters is protected by a polyvinyl alcohol layer. The paint application process leads to differences in the thickness of the paint in different regions, which directly affects the anti-rust performance. The study reveals the deterioration mechanism of resulfurized steel components in a subtropical polluted environment and puts forward repair suggestions that consider both material compatibility and reversibility, providing a reference for the protection practice of modern and contemporary architectural metal heritage in Macau and even in similar geographical environments.

## 1. Introduction

As an important coastal city in China, Macau has a unique historical and cultural background and architectural heritage [1]. Modern and contemporary buildings, especially those during the Portuguese rule, widely use metal components such as iron windows, iron doors, railings, and metal signs [2]. However, due to Macau’s special climate, these metal components are prone to corrosion and degradation under long-term exposure to humidity, heat, high salinity, and air pollution [3]. Therefore, studying the corrosion characteristics and influencing factors of metal components in Macau’s modern and contemporary buildings is not only crucial for the protection of architectural heritage but also has reference value for the durability of metal materials in subtropical coastal environments.

This study selected Vila D. Bosco as the research object. This building is one of the important representatives of the Portuguese rule in Macau. Numerous environmental factors severely corrode its metal components. By analyzing its location, we can deeply explore the potential impact of the external environment on the corrosion of building metal components.

Research on modern and contemporary metal products has had positive impacts on the development of modern industry in many aspects, such as improving material performance [3,4], innovating manufacturing processes [5,6], optimizing product design [7,8], and sustainable industrial development [9]. For example, through the research on modern and contemporary metal products, researchers have continuously explored the relationship between the microstructure and performance of metal materials and developed a series of high-performance metal materials [10]. Some scholars have found that adding specific alloying elements can significantly improve the strength and corrosion resistance of metals, which has led to the widespread use of high-performance alloys, such as stainless steel and aluminum alloys, in aerospace, automobile manufacturing, and other fields [11]. High-strength aluminum alloys used in aerospace, with their lightweight and high-strength characteristics, help aircraft reduce weight and improve fuel efficiency and flight performance. The industry widely uses modern metal products.

In the field of cultural heritage, recent research on modern and contemporary metal products covers many aspects, such as composition analysis, manufacturing process, corrosion, and protection [12,13,14,15,16,17]. However, each cultural heritage has its own uniqueness, and the commonalities of many studies may not be similar. Researchers I. Crina Anca Sandu and others studied old “gilded” artworks from the 9th to 19th centuries in European culture using imaging and spectral analysis methods. They looked into how these artworks are preserved, how they change and age over time, and how restoration treatments like cleaning, strengthening, changing, and glazing affect the condition of the original materials [18]. Other scholars have studied the industrial buildings of steel mills that operated in Slovakia from 1800 to 1948, analyzing their architectural and urban characteristics, evaluating the value of metal products in industrial architectural heritage, and formulating protection strategies based on their characteristics [19]. Researchers Matthew Carl and others found that the makeup, thickness, feel, and state of the coating and the main material work well together by studying the coating and base metal of the American silver-plated cultural heritage collection at the Dallas Museum of Art (DMA) [20]. Iranian scholars have studied 14 Achaemenid bronze artifacts in the Persepolis World Heritage Site (550–330 BC) using methods, such as inductively coupled plasma emission spectroscopy, scanning electron microscopy–energy dispersive spectroscopy, and metallographic analysis, and have identified their manufacturing processes, such as casting, coldworking, and annealing. Similar methods can be used to study modern and contemporary metal products, such as iron components and metal decorative parts in modern and contemporary architectural heritage, to explore the inheritance and development of their production processes, as well as the technological level at the time [21]. Based on existing research, we can use modern analytical technology to obtain detailed information on the composition and microstructure of metal products [22]. For example, through a non-destructive portable X-ray fluorescence spectroscopy (XRF), scanning electron microscope equipped with energy dispersive spectroscopy (SEM/EDS) and other technologies, the alloy composition of ancient metal products can be analyzed, which is also of reference significance for understanding the composition of modern metal products. It can help identify the source of modern metal products’ materials, the proportion of materials used in production, etc., to better protect and study the heritage metal products.

Existing research mostly focuses on the corrosion of industrial facilities and smaller metal objects in the museum collection, and there is little systematic research on metal components of architectural heritage [23]. Vila D. Bosco in Macau is a typical modern building from the Portuguese rule in the 1960s, reflecting the architectural style and cultural background of Portugal in Macau at that time. The villa nameplate not only has decorative and identification functions but also carries important historical and cultural values. However, the current nameplate has serious metal corrosion, the surface coating has fallen off widely, and the metal substrate is seriously corroded. X-ray fluorescence spectroscopy (XRF) analysis did not find any chlorine; therefore, we initially ruled out the typical direct damage from seawater or sea salt. This finding raises a series of research questions:

(1) What is the specific composition of the nameplate metal material and its process characteristics?
(2) In the absence of chlorine, what environmental factors, pollutants, or other chemical mechanisms may have caused such severe corrosion damage?
(3) Do the metal materials and surface coatings used in modern buildings in Macau during the Portuguese rule have specific era characteristics? How do these characteristics interact with local climate, history, and cultural factors to affect the durability of the materials?

This study aims to better understand how metal materials behave and corrode when protecting Macau’s historical buildings, and to offer expert advice for future restoration and protection efforts.

## 2. Materials and Methods

### 2.1. Research Subjects: Vila D. Bosco

Vila D. Bosco (鮑思高別墅) is located along the Estrada de Choc Van (竹灣馬路) on Coloane Island in the Macau Special Administrative Region, facing the South China Sea. The building is situated in a region characterized by a typical subtropical marine climate, with high relative humidity (averaging over 80% annually), frequent rainfall, and strong sea breezes, especially during the summer typhoon season [24]. These climatic conditions are known to significantly influence the weather and corrosion of metal materials.

**Figure 1:**
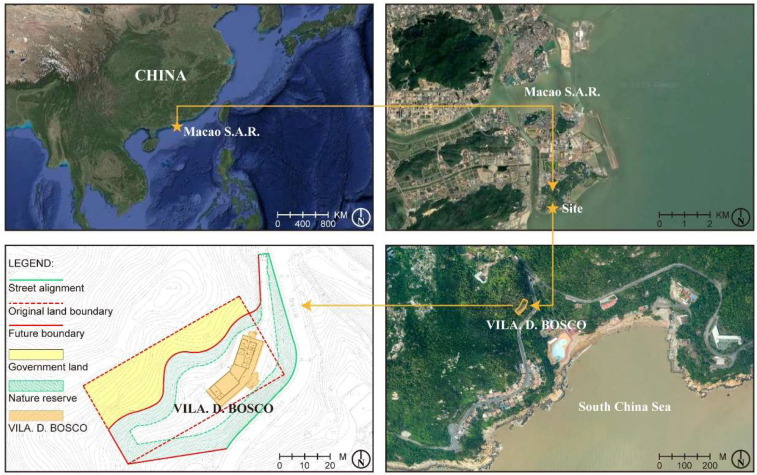
Location and land use conditions of Vila D. Bosco.

The villa is positioned directly adjacent to the South China Sea and is continuously exposed to the marine atmosphere (Figure 1). The coastal environment, with its high wind speeds and air mobility, can facilitate the transport of atmospheric pollutants such as sulfur dioxide (SO_2_) and nitrogen oxides (NO_x_) [25]. In humid conditions, these pollutants may form acidic substances that contribute to the corrosion of exposed metal surfaces [26]. Although prior studies have indicated that sea salt aerosols might not be the primary driver of corrosion, the presence of salt can still exacerbate surface deterioration in marine environments [27,28,29]. Surrounding Vila D. Bosco are extensive nature reserves with dense vegetation cover, which may influence local microclimate factors such as humidity levels [30].

**Figure 2:**
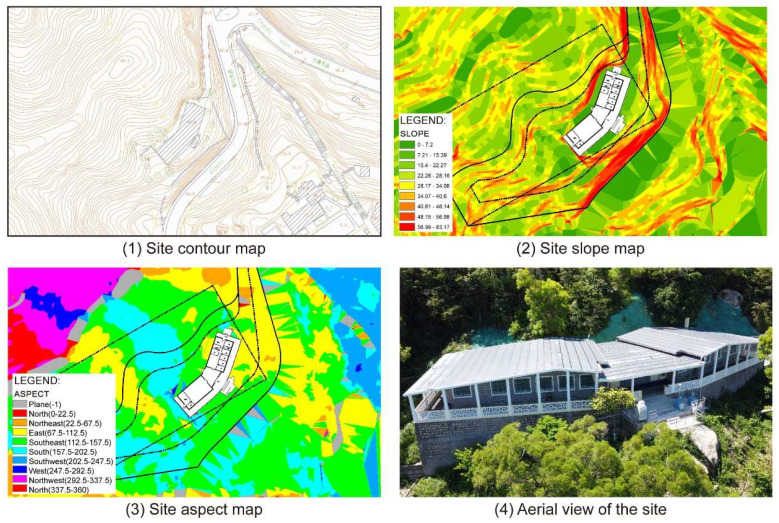
Vila D. Bosco contour lines, aspect, slope, and bird’s eye view.

Topographic analysis shows that Vila D. Bosco is located on a southeast-facing hillside on Coloane Island (Figure 2). As seen in the contour map (Figure 2(1)) and slope analysis (Figure 2(2)), the building sits on terrain with moderate to high slopes (15° to 40°), suggesting a varied microtopography. The main axis of the building follows the contour lines, aligned approximately north–south. Vegetation surrounding the site (Figure 2(4)) provides partial shielding from direct marine aerosol deposition, although it may also affect local humidity dynamics.

**Figure 3:**
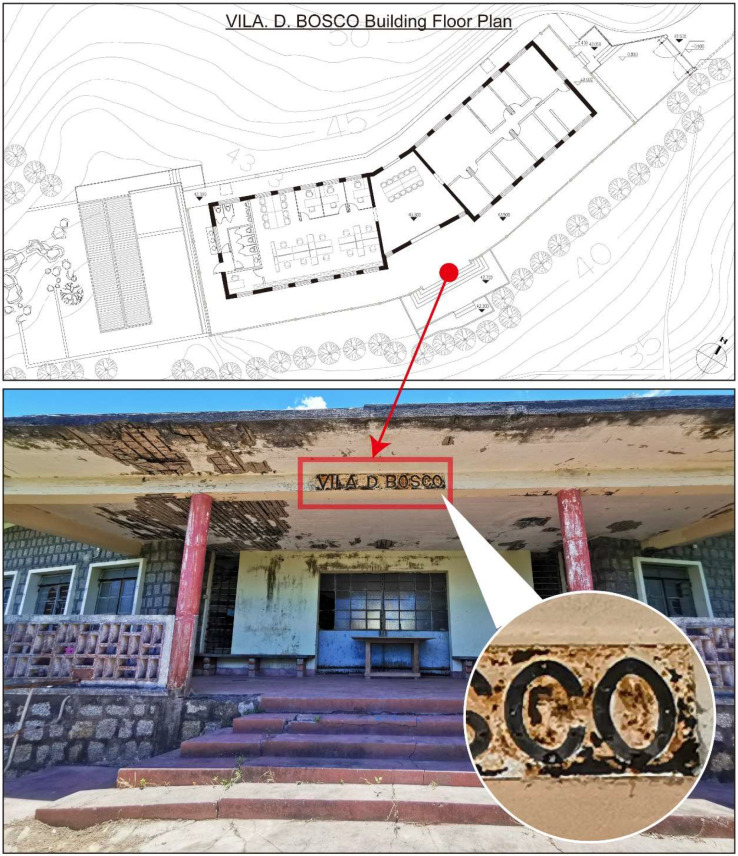
Vila D. Bosco nameplate corrosion status.

Originally built in the 1960s by Salesian priests of Don Bosco College, Vila D. Bosco served as a holiday camp facility for young people during a time when beach vacations in Taipa and Coloane were popular among affluent Macanese residents [31]. After decades of exposure to the elements, the villa’s structure and appearance have deteriorated [32]. Following a restoration project, the site is now managed by the Ministry of Water Resources’ Digital Twin Watershed Key Laboratory. The nameplate of Vila D. Bosco is installed under the eaves at the building’s main entrance (Figure 3), exposed to a semi-open outdoor environment. Field observation revealed extensive rusting, significant paint peeling, and localized pitting on the nameplate surface, indicating advanced stages of material degradation. Similar signs of deterioration, including peeling paint, concrete spalling, and biological staining, were observed on surrounding structural elements, reflecting prolonged exposure to harsh environmental conditions.

**Figure 4:**
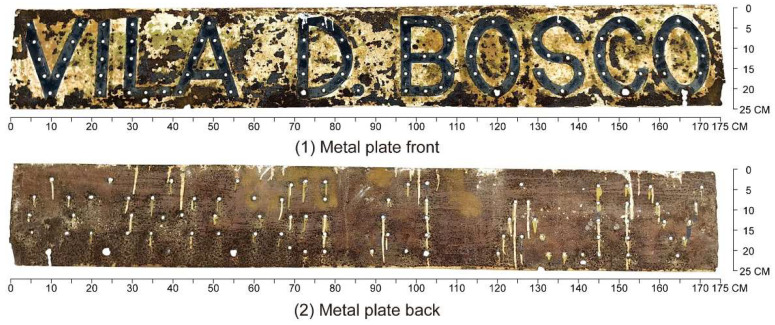
Vila D. Bosco nameplate.

**Figure 5:**
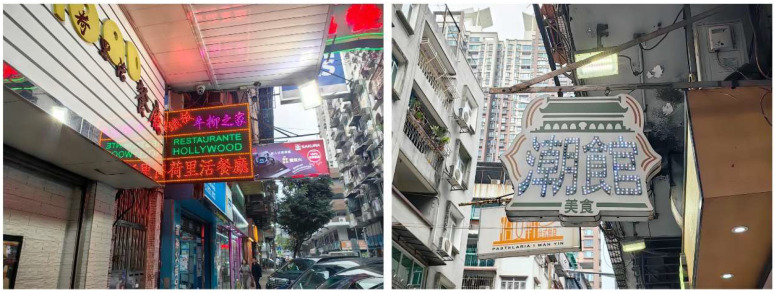
Marquee letters used on restaurant signs on the streets of Macau Peninsula. The Chinese characters in the picture are the names of the signs on the street shops and have no specific meaning.

The nameplate itself is a rectangular metal plate measuring approximately 173 cm in length and 24 cm in width (Figure 4). Its design features marquee-style three-dimensional letters reading “VILA D. BOSCO”, each incorporating sockets for light bulbs. This stylistic approach, popular in early 20th-century signage in Europe and the United States, remains visible in many historical shopfronts within the Macau Peninsula (Figure 5). On the nameplate, the front surface exhibits extensive paint loss, visible rust patches ranging from yellow-brown to dark brown, and isolated greenish stains likely related to the degradation of protective coatings. The back of the nameplate shows more uniform and extensive corrosion, with dark brown rust layers and signs of coating overflow residues. These observations suggest long-term exposure to humid, saline, and pollutant-laden atmospheric conditions.

### 2.2. Analysis Methods and Processes

The Vila D. Bosco nameplate samples analyzed in this study include on-site non-destructive testing of the entire nameplate and laboratory analysis of selective sampling from the nameplate surface. The specific research methods and steps are as follows:

#### 2.2.1. SEM-EDS Micromorphology and Elemental Analysis

The researchers used a scanning electron microscope (SEM, FEI Quanta 250) combined with energy dispersive X-ray spectroscopy (EDS) to observe the micromorphology and analyze the elemental composition of the small samples. During the experiment, the sample was attached to the stage with conductive glue and covered with carbon to help improve the clarity and quality of the images taken by the electron microscope. The SEM operating voltage was set to 20 kV, the beam current was about 0.5 nA, and the working distance was about 10 mm. The EDS analysis time was 60 s per point, and the data obtained was used to determine the element distribution characteristics of the corrosion area and the possible corrosion mechanism [33,34,35].

#### 2.2.2. X-Ray Fluorescence Spectroscopy (XRF) Analysis

A portable X-ray fluorescence spectrometer (Bruker Tracer 5i, Karlsruhe, Germany) was used for on-site testing to directly analyze the elemental composition of the nameplate metal substrate and coating in a non-destructive manner [36,37]. The measurement time for each test point was 120 s; the X-ray tube voltage was set to 40 kV, and the current was 35 µA to obtain qualitative and semi-quantitative information about the elements. Small-sized samples (about 5 mm × 5 mm) were selected from areas with different degrees of corrosion on the nameplate for subsequent laboratory testing and analysis. The collected small samples were washed with anhydrous ethanol and dried in a vacuum drying oven at 60 °C for 24 h for use to avoid interference from external contaminants.

#### 2.2.3. XRD Corrosion Product Phase Analysis

X-ray diffraction (XRD) analysis was performed using the Olympus BTX™ III Benchtop XRD Analyzer to identify the crystalline phases present in the corrosion products on the nameplate [38,39]. The instrument uses a Co-Kα ray source (λ = 1.78897 Å), with the tube voltage set at 30 kV and the tube current at 0.33 mA. The scanning was carried out over a 2θ range from 5° to 55°, with a step size of 0.25°. The researchers used Xpowder™ software (http://www.xpowder.com, accessed on 16 March 2025) to compare the obtained XRD spectrum with the Powder Diffraction File (PDF) database, determining the crystalline phases of the corrosion products [40,41]. Since most reference spectra and mineral standards (e.g., those from the RRUFF project) are based on Cu-Kα radiation (λ = 1.5406 Å), the collected Co-Kα diffraction patterns were converted to their Cu-Kα equivalents using Python (version 3.12) to facilitate accurate phase matching. This wavelength transformation ensures consistency when comparing experimental results to the Powder Diffraction File (PDF) and the RRUFF database. Peak positions were cross-checked with standard patterns from the RRUFF project, and corresponding reference spectrum IDs were noted.

#### 2.2.4. Fourier Transform Infrared Spectroscopy (FTIR) Analysis

The organic components of the coating on the surface of the nameplate were analyzed using attenuated total internal reflectance Fourier transform infrared spectroscopy (ATR-FTIR, Perkin Elmer Spectrum III, PerkinElmer Inc, Waltham, MA, USA) [42]. When sampling, a small amount of the sample (about 1 mg) was taken from the coating part and measured non-destructively using ATR. The scanning range was set to 4000–650 cm^−1^, the resolution was 4 cm^−1^, and the number of scans was 50. The organic components in the coating, such as resins and pigments, were identified by comparing the FTIR spectrum’s characteristic peaks with the standard spectral library.

#### 2.2.5. Cross-Section Microscopic Analysis

Four samples of about 2 mm in length and 1 mm in width (from the surface of the paint to the base of the nameplate) were removed from the white and black paint on the nameplate. These samples were placed in a mold, and Buehler™ epoxy resin was dripped into the mold. The samples were then left to stand and polished. Finally, the sample that has been polished to expose the cross-section is placed under a Motic M230T (Motic Asia, Kowloon, Hong Kong) optical microscope to observe the sample cross-section at a magnification of 50×.

Using the detailed experimental methods mentioned above, it can effectively determine the elemental makeup, microstructure, and crystal structure of rust products, as well as the organic content of the nameplate metal material, allowing for a thorough analysis of the underlying material science reasons for the nameplate corrosion issue.

## 3. Results

### 3.1. SEM-EDS Microstructure and Elemental Composition Analysis

**Figure 6:**
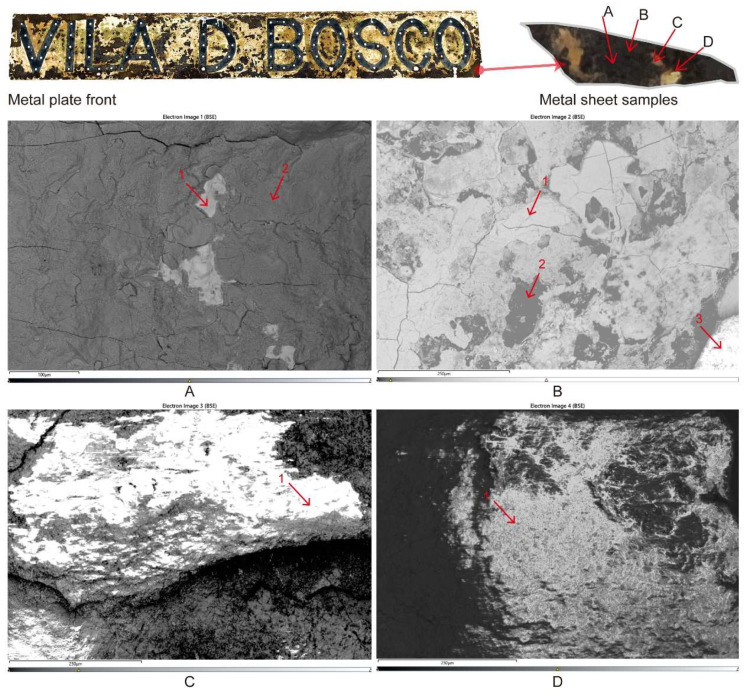
SEM micrographs of different regions (**A**–**D**) of the Vila D. Bosco nameplate and sampling points for EDS analysis.

The microscopic morphology of different areas of the Vila D. Bosco nameplate was observed using scanning electron microscopy (SEM) combined with energy dispersive spectroscopy (EDS). As shown in Figure 6, multiple typical corrosion features were identified, such as rust layers, micro-cracks, and layered flake structures. To ensure the reliability and representativeness of the analysis, three independent EDS measurements were conducted for each selected region (A–D), and the results were averaged and summarized in Table 1.

In sample A (Figure 6A), the surface shows relatively limited corrosion. EDS point 1 indicates a high iron (Fe) content (95.58%) and a low oxygen (O) content (3.08%), suggesting the presence of the exposed metallic substrate. Points 2 and 3 reveal slight increases in oxygen (19.85% and 5.66%, respectively), accompanied by minor amounts of sulfur (0.58% at point 2) and cobalt, reflecting the initial stages of oxide formation and environmental deposition.

In sample B (Figure 6B), the corrosion features are more prominent. EDS results show that oxygen content varies significantly from 39.91% to 54.53%, while iron content drops to between 26.89% and 46.42%. High concentrations of silicon (up to 10.87%), sulfur (up to 9.29%), potassium, and zirconium were also detected. These findings suggest sulfate corrosion and possible environmental dust deposition. The considerable presence of sulfur and other non-metallic elements indicates that this area is particularly vulnerable to chemical attack under humid and polluted atmospheric conditions.

Sample C (Figure 6C) mainly represents areas associated with paint residues and corrosion layers. The EDS points reveal an oxygen content around 31.79–38.09%, with substantial enrichment in zinc (up to 19.50%), calcium (up to 21.56%), and sulfur (up to 11.96%). Trace levels of titanium, aluminum, nickel, and chromium were also detected. This elemental composition implies that this area includes residues from zinc-rich coatings and the accumulation of environmental dust. The combination of sulfur and calcium suggests the possible formation of sulfate salts on the surface.

Sample D (Figure 6D) exhibits a complex, highly deteriorated morphology. EDS results indicate oxygen contents between 36.49% and 38.79%, and iron levels between 26.92% and 31.45%. Sulfur remains significantly high (7.28–12.78%), along with considerable amounts of zirconium, potassium, titanium, aluminum, and silicon. These results confirm the formation of corrosion products mainly composed of iron oxides and sulfate compounds, combined with environmental pollutant deposition. Severe coating peeling and corrosion-product accumulation were observed in this area.

Overall, the SEM-EDS analysis confirms that the Vila D. Bosco nameplate has undergone multiphase corrosion processes, characterized by localized oxidation, environmental particle deposition, and surface deterioration associated with complex atmospheric exposure. Due to equipment limitations, the SEM images shown reflect the maximum achievable resolution during testing.

**Figure 7:**
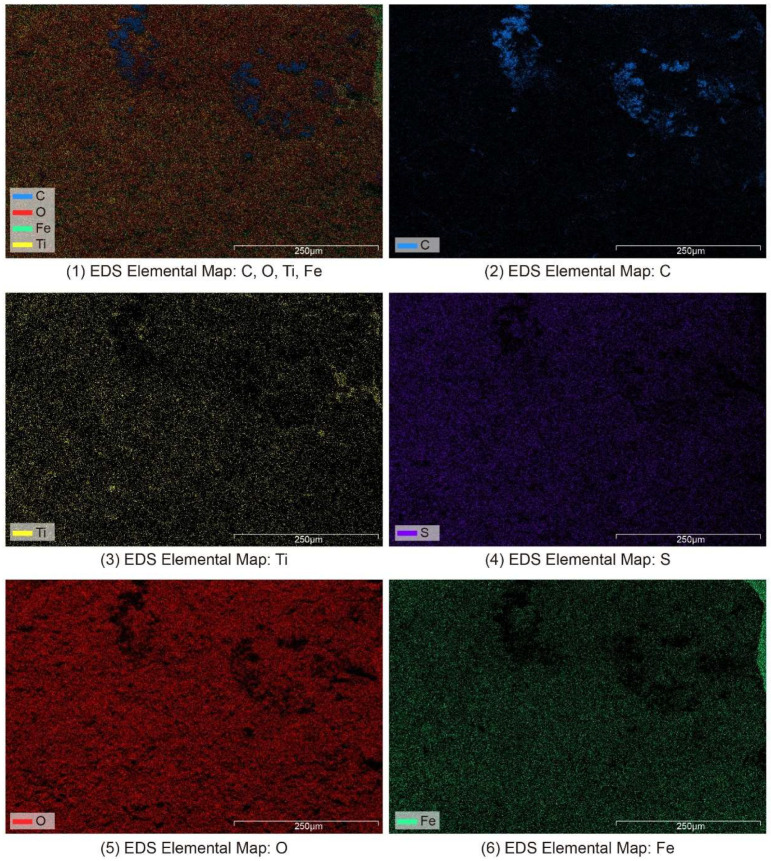
SEM-EDS elemental surface scanning image of the Vila D. Bosco nameplate.

In this study, an elemental surface scan (Figure 7) was performed on sample D (Figure 6D). The carbon, oxygen, titanium, and iron in the nameplate were evenly distributed, indicating that the nameplate was oxidized to form evenly distributed rust. Carbon (C) was found in higher amounts in certain spots (Figure 7, blue area), likely due to the buildup of organic materials in the coating (like alkyd resin or polyvinyl alcohol) and organic dirt from the outside environment. This finding further confirms the significant effect of aging and shedding of the coating on the element distribution in the corrosion area of the nameplate. The titanium element should come from the residue of titanium dioxide pigment in the coating. It is worth noting that the bright area in sample D is white paint, which should be rich in titanium; however, its EDS analysis results indicate that the titanium content in the bright area is not much different from that in the dark rust area. It is speculated that the weak conductivity of the coating itself causes the weak EDS signal. In addition, the distribution of sulfur elements throughout the surface of the nameplate is closely related to the fact that the nameplate’s original material is resulfurized steel. A certain proportion of sulfur is added to resulfurized steel during the smelting process to improve the material’s cutting performance. However, in a humid subtropical environment, this type of steel is more likely to rust due to sulfur, leading to a large increase in sulfur in the rust. It should be noted that although the carbon detected in the SEM-EDS surface analysis was primarily attributed to external organic contamination (such as aged coatings and environmental deposits), due to the limited sensitivity of EDS to light elements like carbon, a small amount of intrinsic carbon from the metal matrix cannot be entirely excluded. Based on the comprehensive analysis of iron and sulfur content and historical background, the base material is more consistent with resulfurized steel rather than raw iron (pig iron) [43].

### 3.2. XRF Elemental Analysis

**Figure 8:**
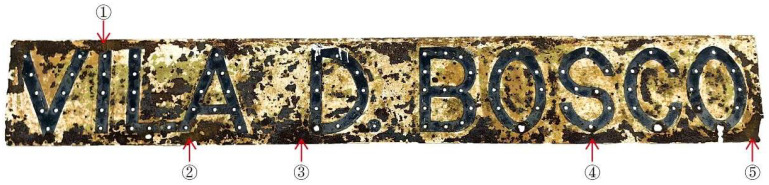
Five detection points of Vila D. Bosco nameplate iron-based XRF analysis.

In order to further clarify the overall chemical composition of the metal matrix of the nameplate and support the conclusion of the SEM-EDS analysis, this study conducted XRF tests on 5 points in the front of the nameplate (Figure 8 and Table 2). The test results indicate that the main component of all samples is iron (Fe), with a mass fraction ranging from 96.34% to 98.22% and an average of 97.30%, indicating that the nameplate is an iron alloy. The Fe content fluctuates slightly between different points; however, it is maintained at a high level overall, indicating that the overall material of the nameplate is relatively uniform, and there is no material replacement due to later maintenance or replacement. A certain proportion of sulfur (S) was detected in all samples, with a content ranging from 0.67% to 1.98%. The content in sample 4 was the highest, significantly higher than the other points. This sulfur content is much higher than the impurity content of sulfur in general low-carbon steel. Combined with the phenomenon of widespread sulfur enrichment in the rusted area in the aforementioned SEM-EDS results, it is speculated that the nameplate uses resulfurized steel. Resulfurized steel was common in industrial products in the mid-20th century and was widely used in nameplates, signboards, and other precision-processed metal components due to its excellent cutting performance [44,45]. However, high sulfur content also reduces the corrosion resistance of steel. In a humid, high-humidity, and pollutant-rich atmosphere, it is more likely to generate sulfate corrosion products, thereby accelerating metal surface oxidation and spalling.

All samples contained trace amounts of silicon (Si), manganese (Mn), copper (Cu), aluminum (Al), and molybdenum (Mo). The contents of Si and Mn were slightly higher, ranging from 0.65–1.13% and 0.34–0.39%, respectively. These elements may be deoxidizers and alloys added during the steel manufacturing process, which can improve the structural stability and strength of the steel to a certain extent. The contents of Cu, Al, and Mo are all at trace levels, which may be impurities in the raw materials, residual pollutants on the surface, or interference from coating components. The nameplate’s corrosion morphology shows that the presence of alloying elements has not significantly changed the material’s overall corrosion resistance.

**Figure 9:**
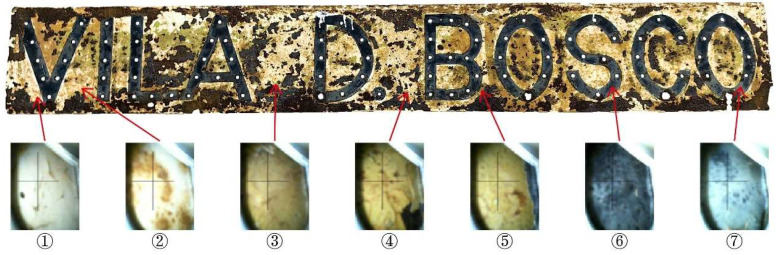
Seven detection points of XRF analysis in the coating on the Vila D. Bosco nameplate.

To determine the material composition and pigment source of the nameplate surface coating [46,47], XRF elemental analysis was performed on a total of 7 points of white, yellow-green, and black coatings on the nameplate (Figure 9 and Table 3).

(1) White coating area (samples 1–2). Samples 1 and 2 mainly consist of titanium (Ti), with amounts of 84.70% and 54.47%, respectively, showing that titanium dioxide (TiO_2_) is used as a color pigment in the white coating. Titanium dioxide has excellent hiding power and weather resistance and is widely used in modern buildings and industrial products. Also, a little bit of aluminum (Al), chromium (Cr), and tantalum (Ta) were found here, likely from impurities or additives in the pigment. Since the white coating has a strong hiding power, XRF cannot detect the metal matrix; therefore, the iron (Fe) content in this area is low.
(2) Yellow-green coating area (samples 3–5). The elemental composition of this area is relatively complex and should be a mixture of multiple color pigments. In addition to the medium contents of Ti (4.56–17.92%) and Fe (15.60–46.30%), relatively high contents of Cr (7.12–12.13%), Pb (12.84–47.27%), and Zn (5.68–12.11%) were also detected. This indicates that the yellow-green pigment is most likely chrome yellow (PbCrO_4_) and zinc yellow (ZnCrO_4_). The mid-20th century saw widespread use of this type of pigment in industrial and architectural coatings due to its strong durability and corrosion resistance. This paint is more common in the metal parts of Portuguese colonial buildings, which is consistent with the nameplate’s production background.
(3) Black coating area (samples 6–7). The Ti content in the black area is still high (54.81% and 60.56%), indicating that the black paint may also be mixed with TiO_2_ as a covering filler. The Fe content is moderate (32.31–39.65%), which matches what we expect from common iron black pigments (like iron manganese oxides); samples 5 and 6 also showed some sulfur (6.55–11.78%), which could come from sulfur-containing black organic or inorganic pigments, or it might be left over from environmental pollution.

### 3.3. XRD Phase Analysis of Corrosion Products

To further investigate the mineral composition and crystalline structure of the corrosion layer on the nameplate, an X-ray diffraction (XRD) analysis was conducted on rust samples collected from its surface. The experiment employed a Co-Kα radiation source (λ = 1.78897 Å), and the resulting spectra were converted to Cu-Kα equivalent values (λ = 1.5406 Å) to allow direct comparison with standard reference data.

**Figure 10:**
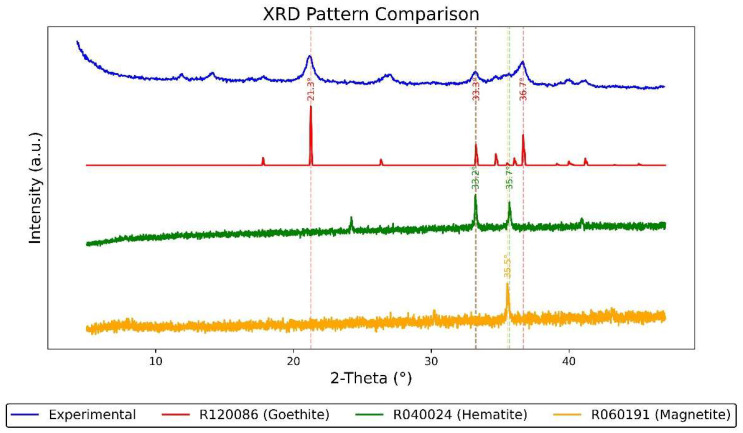
XRD analysis results of rust on the Vila D. Bosco nameplate.

As shown in Figure 10, the diffraction pattern of the experimental sample (converted to Cu-Kα) is compared with reference spectra from the RRUFF database. Specifically, goethite (RRUFF ID: R120086), hematite (RRUFF ID: R040024), and magnetite (RRUFF ID: R060191) are included in the comparison. Major peak positions are annotated based on this matching process. The experimental spectrum shows several distinct peaks at approximately 2θ ≈ 21.3°, 33.3°, 36.7°, etc. Notably, the peaks around 33.2° and 35.5° are characteristic of hematite (α-Fe_2_O_3_) and magnetite (Fe_3_O_4_), confirming the presence of these phases. A peak at 21.3°, corresponding to goethite (α-FeOOH), further suggests a typical mixed iron oxide rust layer. This combination of phases indicates that the corrosion developed in a humid, oxygen-rich environment, with possible exposure to mildly acidic airborne particles—a scenario consistent with the subtropical climate of southern Macau. The detection of stable phases like goethite implies that the corrosion occurred under alternating wet and dry conditions, allowing for both active oxidation and passive stabilization processes over time.

**Figure 11:**
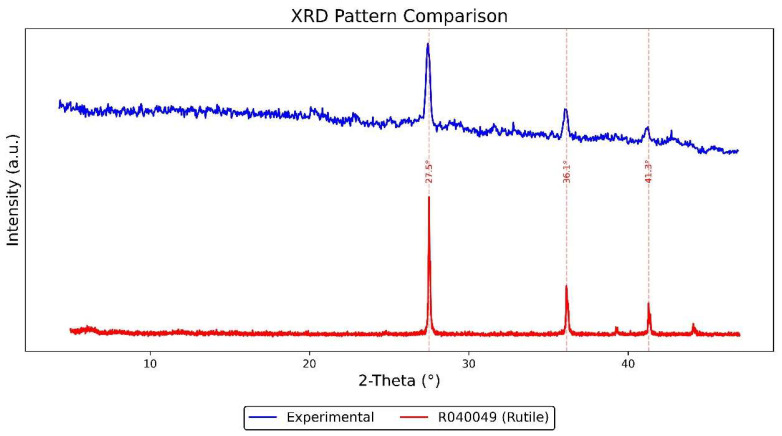
XRD analysis results of the Vila D. Bosco nameplate’s white coating.

The XRD results of the white coating (Figure 11) indicate that the sample predominantly consists of synthetic rutile (TiO_2_, RRUFF ID: R040049). The experimental pattern—converted from Co-Kα to Cu-Kα radiation—shows strong diffraction peaks at 2θ ≈ 27.5°, 36.1°, and 41.3°, which align closely with the reference peaks of rutile. These results confirm that the white pigment is crystalline and primarily composed of TiO_2_ in its rutile form, commonly used in industrial coatings as a white pigment.

In contrast, the XRD analysis of the black and yellow-green coatings on the nameplate revealed broad background signals and baseline drift, with no discernible sharp diffraction peaks. This suggests that these layers are either amorphous or possess an extremely low degree of crystallinity. Such results are typical of complex or degraded organic coatings or corrosion products where crystalline phases are poorly developed.

### 3.4. FTIR Spectroscopy Analysis of Coatings

**Figure 12:**
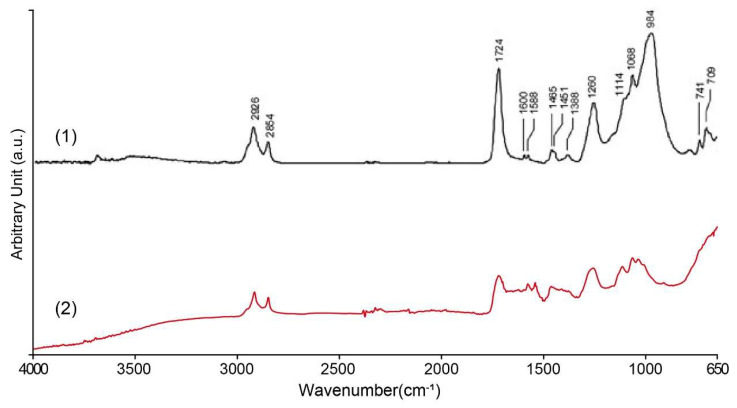
FTIR analysis of white and yellow-green coating on the Vila D. Bosco nameplate: (1) alkyd binder in mixture with inorganic pigments [48]; (2) the results of this study.

In order to identify the organic components in the surface coating of the nameplate, ATR-FTIR tests were performed on white, yellow-green, and black coating samples. Both white and yellow-green coatings contain alkyd resin (polyester alkyd), and the comparison with the standard spectrum is shown in Figure 12. The sample shows a strong C–H stretching vibration absorption peak at ~2925 cm^−1^, corresponding to the vibration of the –CH_2_– (methyl) group of the aliphatic hydrocarbon chain, indicating that the sample contains a segment of a long fatty acid chain. The strong absorption peak near 1730 cm^−1^ is the C=O carbonyl stretching vibration of the ester, which is a typical feature of alkyd, polyester, or other ester-based resins. Additionally, a faint peak at 1638 cm^−1^, which may be attributed to C=O stretching and/or C=C stretching vibrations, is consistent with the typical spectral features of alkyd resins. Several absorption peaks were observed in the 1150–1000 cm^−1^ region, which can be attributed to C–O–C and C–O stretching vibrations. These peak shape characteristics are also consistent with the alcohol ester structure of alkyd paint. There is also a C–H out-of-plane bending vibration peak at approximately 720–740 cm^−1^, indicating the presence of some hydrocarbon side chains in the sample.

**Figure 13:**
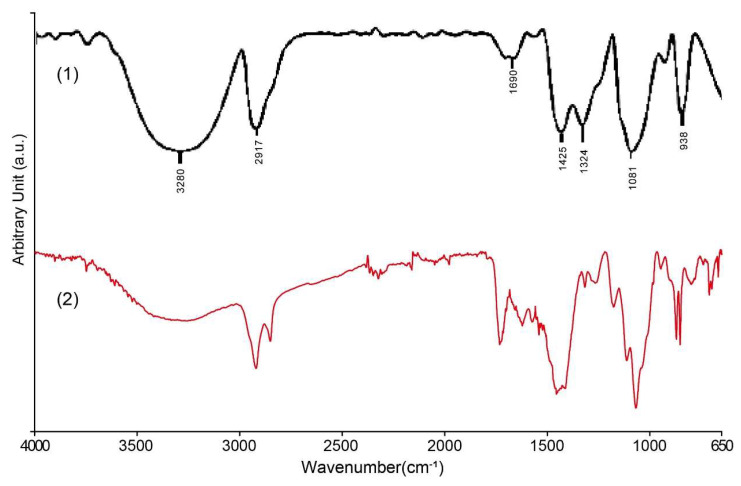
FTIR analysis of black coating on the Vila D. Bosco nameplate: (1) PVA [49]; (2) the results of this study.

The FTIR spectrum results of the black coating sample show that its main component is polyvinyl alcohol (Figure 13). The broad and strong absorption peak at about ~3300 cm^−1^ in the spectrum corresponds to the stretching vibration of the hydroxyl group (–OH); the absorption peak near about 2920 cm^−1^ corresponds to the asymmetric stretching vibration peak of the methylene group (–CH_2_–) in the aliphatic chain segment; the weak peak at about 1700 cm^−1^ should be attributed to the stretching vibration peak of the carbonyl group (C=O) contained in the small amount of polyvinyl acetate (PVAc) component in the hydrolysis process of the residual polyvinyl alcohol in the sample. In addition, the strong peak at about 1080 cm^−1^ shows the stretching vibration of the alcohol group (C–O) in the polyvinyl alcohol chain. These absorption peaks match very well with the known features of standard polyvinyl alcohol, confirming that the black coating material is indeed polyvinyl alcohol. At the same time, due to incomplete hydrolysis during the preparation process, a small amount of polyvinyl acetate components remains in the coating. The significant differences in elemental composition between the coatings and the metal substrate suggest that the substrate was initially painted, and the decorative letters were installed afterward.

### 3.5. Cross-Section Microscopic Analysis

**Figure 14:**
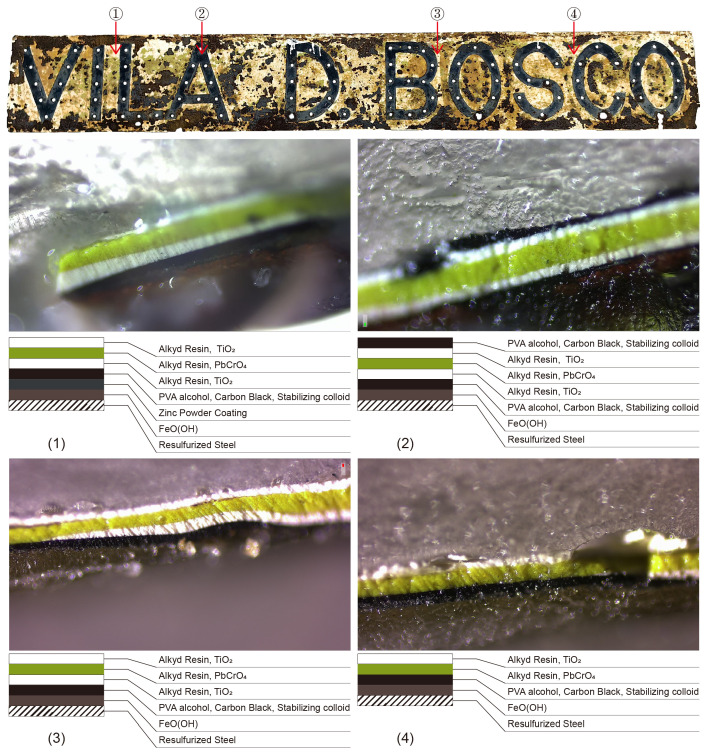
Microscopic observation of the cross-section of the Vila D. Bosco nameplate.

The microphotographs (Figure 14) show that the cross-sections of the four Vila D. Bosco nameplate coating samples have different numbers of layers; however, they all have a white-green-white overlay structure. The compositional interpretation of the observed layers is based on the correlation of morphological features with the results from XRD and FTIR analyses, rather than the microscopic images alone. Sample 1 is located on a white background, with titanium white resin paint on the top layer, a yellow-green lead yellow resin paint on the second layer, titanium white paint on the third layer, black paint on the fourth layer, a zinc powder layer on the fifth layer, and a rust layer on the sixth layer. Sample 2 features black letters, black paint on the top layer, titanium white resin paint, lead yellow resin paint, titanium white resin paint, black paint, and a rust layer underneath. Sample 3 is like sample 2, but it has a white background and no black surface layer. In the microphotograph of sample 4, there is no titanium white coating above the rust layer, but a small amount of it can be observed on the right side of the picture. We speculate that either the paint application was uneven or the paint was loose during production, leading to the white layer falling off.

## 4. Discussion

Based on the results of XRF, SEM-EDS, XRD, ATR-FTIR, and cross-section microscopic analysis, this study systematically revealed the material composition, corrosion mechanism, and surface coating structure characteristics of the Vila D. Bosco nameplate.

XRF testing shows that the nameplate is an iron alloy with an iron content of 96–98% and about 0.7–2.0% sulfur (S). Combined with its historical background and the application characteristics of common metal materials in the mid-20th century, it is speculated that the nameplate is made of resulfurized steel. This type of steel can improve cutting performance due to its sulfur content and is often used for precision-processed components such as nameplates. However, its high sulfur content also makes it easy to generate sulfate corrosion products in hot and humid atmospheres, thereby reducing corrosion resistance.

SEM-EDS and XRD analysis show that the nameplate’s rusted area is rich in Fe, O, S, Ti, and Zn. The main types of rust found are goethite (α-FeOOH), hematite (α-Fe_2_O_3_), and magnetite (Fe_3_O_4_), which are common results of rusting in the air and are usually seen in Macau’s humid, salty, and polluted environment.

FTIR testing confirmed that the white and yellow-green coatings on the nameplate were oil-modified alkyd resin pigments. XRF and XRD tests showed that the coating used synthetic rutile titanium dioxide, chrome yellow, zinc yellow, and other pigments, which together made a strong industrial coating that hides well and lasts outdoors. Unlike the matte white background, the black coating of the letters has a glazed gloss, and the FTIR results are polyvinyl alcohol. Since polyvinyl alcohol is rarely used directly as a pigment binder, it is speculated that polyvinyl alcohol is a transparent protective layer on the surface of the black coating, and the binder of the black coating itself is unknown.

The cross-sectional microscopic analysis of the samples indicated that the nameplate coating method was to apply black paint as a primer on the zinc powder coating, then apply it on the black base layer in the order of white-green-white, and finally draw black letters on the white background layer. Only the bottom layer of sample 1 displayed the presence of the zinc powder layer among the four samples. The reasons for this phenomenon are as follows: (1) the zinc coating layer separated from the surface coating after deterioration; (2) considering that there was no gap between the titanium dioxide layer and the rust layer in samples 2, 3 and 4, the initial process may have been immature and caused part of the nameplate surface to be ungalvanized. The coating thickness in the samples was uneven, the surface was not smooth, and the titanium dioxide layer was relatively thick in some places, which also indicated that a spray gun was not used during production, but was painted by hand. The uneven surface coating made the anti-rust performance of the nameplate surface uneven, resulting in varying degrees of deterioration. In particular, the lack of the zinc powder layer weakened the cathodic protection of the alloy matrix. Since the resulfurized steel is prone to corrosion from sulfur, some areas in a wet and polluted environment made the problem worse and allowed it to spread.

After systematically analyzing the metal material, corrosion products, coating composition, and layered structure of the Vila D. Bosco nameplate, one of the ultimate goals of this study is to provide a scientific, operational, and academically based protection and restoration strategy for this type of modern architectural metal component. The nameplate material is mainly resulfurized steel, the corrosion products are mainly mixed iron oxides, the white coating is TiO_2_ alkyd resin paint, and the yellow-green coating is PbCrO_4_ alkyd resin paint. From the perspective of material chemistry, the deterioration mechanism it faces includes not only typical atmospheric oxidation corrosion, but also local failures caused by interlayer peeling, pigment aging, and uneven original construction. Relying solely on a single material or restoration process cannot achieve long-term, stable protection for these different types of problems. A “multi-material-multi-mechanism” collaborative restoration method must be adopted to consider the structural, functional, and cultural expression integrity.

Therefore, combined with the experimental results and cross-sectional structure analysis in the previous article, this paper summarizes a comprehensive protection plan including rust removal, functional material recoating, organic layer reconstruction, and reversible sealing, and the summary is shown in Table 4. Each technical treatment suggestion clearly points to the specific problem identification basis and selects modern materials with good compatibility based on the material properties. For example, in response to the problem of partial loss of the zinc powder protective layer, it is recommended to use high-content zinc-rich epoxy primer to restore its cathodic protection ability; for the reconstruction of the multi-layer organic alkyd resin structure, it is recommended to give priority to alkyd resin with a highly similar chemical structure to the raw material and strictly control the PVC value of the pigment to ensure its visual restoration and material stability [50,51]. In addition, for the repair of the blurred area of the nameplate text, a non-invasive micro-brush finishing process should be adopted as much as possible, supplemented by a standard method for controlling color differences to ensure that the historical information of the text is accurately restored. At the same time, all repair measures follow the principle of “reversibility” and try to avoid irreversible reactions and material cross-linking in the selection of bonding materials, coating components, and construction processes to ensure the flexibility of sustainable maintenance in the future. The entire restoration system advocates a strategy of zoning treatment and adapting to local conditions. Different treatment measures should be applied to different structural parts and degrees of deterioration, rather than integrated coverage, to respect the continuity of the historical layered information of the original components.

## 5. Conclusions

This study systematically investigated the material characteristics and degradation mechanisms of the Vila D. Bosco nameplate, a representative metal component from modern and contemporary architecture in Macau during the Portuguese rule in the 1960s. Through a combination of XRF, SEM-EDS, XRD, FTIR analyses, and cross-sectional microscopy, the following conclusions were drawn:

(1) Metal Matrix Composition: The nameplate substrate is composed of resulfurized steel with an iron (Fe) content of up to 97% and sulfur (S) content between 0.7 and 2%. The high sulfur content contributes significantly to internal sulfate corrosion under humid and polluted atmospheric conditions.
(2) Corrosion Products: The main corrosion products include goethite (α-FeOOH), hematite (α-Fe_2_O_3_), and magnetite (Fe_3_O_4_), forming a multiphase mixed structure indicative of prolonged and progressive corrosion processes.
(3) Coating Materials: The surface coatings were identified as based on alkyd resin, incorporating pigments such as titanium dioxide (TiO_2_), lead chromate (PbCrO_4_), and zinc chromate (ZnCrO_4_), reflecting typical mid-20th-century material usage.
(4) Layered Degradation: Cross-sectional analysis revealed significant stratification, including complete coating structures with zinc-rich primer layers and areas of coating disorder caused by aging, maintenance interventions, and environmental factors.
(5) Based on these findings, this study proposes conservation strategies emphasizing material compatibility, structural integrity restoration, and reversibility. The outcomes provide scientific guidance for the protection of similar modern metal components, balancing structural functionality with cultural significance.

## Figures and Tables

**Table 1:** Elemental composition results of the Vila D. Bosco nameplate surface based on EDS point analysis (average of three points per sample).

Element	Sample A [%]			Sample B [%]			Sample C [%]			Sample D [%]		
	1	2	3	1	2	3	1	2	3	1	2	3
O	3.08	19.85	5.66	39.91	50.66	54.53	38.09	32.18	31.79	38.36	38.79	36.49
Mg	0.00	0.00	0.00	0.00	0.82	3.06	0.95	1.59	0.98	0.00	0.00	0.00
Al	0.13	0.00	0.18	1.81	0.30	5.84	4.19	2.27	2.74	2.42	4.11	4.46
Si	0.00	0.00	0.00	10.87	0.36	3.95	0.00	2.15	3.51	2.51	4.88	5.47
S	0.00	0.58	0.00	9.29	0.32	1.18	11.96	5.00	3.34	12.78	11.03	7.28
K	0.00	0.00	0.00	4.13	0.00	0.34	0.72	0.00	1.22	4.54	4.30	2.55
Ca	0.00	0.00	0.00	0.27	0.38	2.43	16.94	5.62	21.56	0.00	0.62	1.62
Ti	0.00	0.00	0.00	3.02	0.16	0.00	4.59	3.39	1.37	3.99	3.13	4.77
Cr	0.00	0.00	0.00	0.00	0.00	0.00	1.26	4.62	0.85	0.00	0.00	0.00
Fe	95.58	78.64	92.86	26.89	46.42	28.67	0.00	14.92	18.77	26.92	27.50	31.45
Co	1.21	0.93	1.30	0.00	0.58	0.00	0.00	0.00	0.68	0.00	0.00	0.00
Ni	0.00	0.00	0.00	0.00	0.00	0.00	1.80	0.78	0.00	0.77	0.00	0.70
Zn	0.00	0.00	0.00	0.00	0.00	0.00	19.50	15.01	6.12	0.00	0.00	0.00
Zr	0.00	0.00	0.00	3.81	0.00	0.00	0.00	12.47	7.07	7.71	5.64	5.21

**Table 2:** Vila D. Bosco nameplate iron-based XRF analysis.

Element Symbol	Value (%)				
	Point ①	Point ②	Point ③	Point ④	Point ⑤
Fe	98.02	98.22	97.80	96.34	98.13
S	0.85	0.70	0.78	1.98	0.67
Si	0.65	0.67	0.92	1.13	0.76
Mn	0.37	0.34	0.39	0.35	0.37
Cu	0.05	0.03	0.04	0.09	0.04
Al	0.03	0.00	0.04	0.08	0.00
Mo	0.03	0.04	0.03	0.03	0.03

**Table 3:** XRF analysis of the coating on the Vila D. Bosco nameplate.

Element Symbol	Value (%)						
	Point ①	Point ②	Point ③	Point ④	Point ⑤	Point ⑥	Point ⑦
Ti	84.70	54.47	10.02	17.92	4.56	54.81	60.56
Fe	8.19	37.90	15.60	46.30	34.39	39.65	32.31
Cr	3.58	3.77	11.92	7.12	12.13	1.17	2.03
Al	1.87	1.45	0.00	1.17	2.07	0.64	1.05
Ta	1.13	0.71	0.00	0.00	1.44	0.32	0.64
Si	0.00	1.34	1.41	1.03	0.06	3.26	3.13
W	0.53	0.30	0.00	0.00	4.59	0.15	0.27
V	0.00	0.05	1.66	1.00	3.14	0.00	0.00
Zn	0.00	0.00	12.11	5.68	0.00	0.00	0.00
Pb	0.00	0.00	47.27	12.84	24.32	0.00	0.00
S	0.00	0.00	0.00	6.55	11.78	0.00	0.00
P	0.00	0.00	0.00	0.40	1.53	0.00	0.00

**Table 4:** Recommendations for the preservation and restoration of the Vila D. Bosco nameplate.

Repair Projects	Problem Identification Basis	Materials/Methods Recommendations	Technical Description
Surface rust removal	The presence of corrosion products such as FeOOH and Fe_2_O_3_, and the rust layer is relatively thick	Use a neutral pH chelating agent (such as EDTA-2Na solution) + mechanical micro-grinding	Avoid using acidic cleaning agents to prevent aggravation of corrosion reaction on resulfurized steel; micro-grinding is limited to non-inscription font areas
Zinc powder protective layer re-coating	The zinc powder layer is partially missing, and the cathodic protection fails	Apply high-purity zinc powder epoxy primer (≥95% Zn content)	Two-component zinc-rich primer can be used to enhance the anti-corrosion performance of the substrate and rebuild the structure of the original zinc powder layer
Black base layer reconstruction	The PVA alcohol + carbon black interface layer is a key stable structure, and it is partially peeled off	Self-prepared polyvinyl alcohol/carbon black dispersion material or commercially available stable carbon black paint	Surface tension and adhesion need to be adjusted to match the original coating thickness (recommended to be controlled at 20–30 μm)
Multi-layer organic paint repair	The organic coating peels off, the layers are uneven, and the light aging is serious	Use alkyd resin adhesive (optionally alkyd resin) + TiO_2_/PbCrO_4_ simulated coating	Apply in layers according to the original structure, first yellow layer and then white layer, and pay attention to controlling the pigment volume fraction (PVC) to ensure hiding power and weather resistance
Protective transparent coating	After aging, the coating has strong hygroscopicity, the surface is slightly cracked, and it needs to be protected from moisture and UV	Apply UV-stable acrylic sealant or fluorocarbon transparent protective film	It is recommended to use products with UV aging resistance of QUV-B 5000h (Q-Lab, Westlake, OH, USA) or above, and control the thickness to 5–10 μm to avoid excessive interference with the apparent color
Nameplate text regeneration	The paint on the edge of the font is seriously peeled off, and some letters are blurred	Use micro-pen repair method + UV-visible color difference comparison and refinement	Fonts are simulated with original black pigment (PVA + carbon black), and redrawn with fine templates when necessary
Reversible repair logo	Ensure the operability of later repairs	Use reversible adhesive for all repair materials, and fully record the repair position	Material records must include brand, ratio, batch number, and use date to meet the principle of reversible protection

## Data Availability

The original contributions presented in this study are included in the article. Further inquiries can be directed to the corresponding author.
